# Data Provenance in Healthcare: Approaches, Challenges, and Future Directions

**DOI:** 10.3390/s23146495

**Published:** 2023-07-18

**Authors:** Mansoor Ahmed, Amil Rohani Dar, Markus Helfert, Abid Khan, Jungsuk Kim

**Affiliations:** 1 ADAPT Centre, Innovation Value Institute, Maynooth University, W23 F2H6 Maynooth, Ireland; markus.helfert@mu.ie; 2 Department of Computer Science, COMSATS University, Federal Capital, Islamabad 44000, Pakistan; amil.rohani@uokajk.edu.pk; 3 Department of Computer Science & Information Technology, Faculty of Computing & Engineering, University of Kotli, Azad Jammu and Kashmir, Kotli 11100, Pakistan; 4 College of Science and Engineering, University of Derby, Derby DE22 1GB, UK; a.khan3@derby.ac.uk; 5 Department of Biomedical Engineering, Gachon University, Seongnam-si 13120, Republic of Korea; 6 Research Institute, Cellico Company, Seongnam-si 13449, Republic of Korea

**Keywords:** data provenance, healthcare, provenance technologies, cryptography, ontologies, blockchain

## Abstract

Data provenance means recording data origins and the history of data generation and processing. In healthcare, data provenance is one of the essential processes that make it possible to track the sources and reasons behind any problem with a user’s data. With the emergence of the General Data Protection Regulation (GDPR), data provenance in healthcare systems should be implemented to give users more control over data. This SLR studies the impacts of data provenance in healthcare and GDPR-compliance-based data provenance through a systematic review of peer-reviewed articles. The SLR discusses the technologies used to achieve data provenance and various methodologies to achieve data provenance. We then explore different technologies that are applied in the healthcare domain and how they achieve data provenance. In the end, we have identified key research gaps followed by future research directions.

## 1. Introduction

The definition of data provenance was introduced by authors [1] many years ago. Various domains have been used for data provenance such as E-services, artificial intelligence, and healthcare. Data provenance is suitable for tracing data history. In healthcare services, data provenance is highly important [2]. We studied the different usage of data provenance by authors in various domains such as [3,4,5,6,7,8,9,10,11,12,13]. In the past, the management and recording of provenance information used to be a manual process. However, due to the sheer volume of provenance information, manual handling of these tasks has become challenging. Scientists and engineers now face significant challenges in both data management and recording provenance information, even for basic inquiries.

In existing research, data provenance is achieved with the help of different technologies. These are log-based [14,15], cryptography-based [16,17,18], blockchain-based [19,20,21], and ontology-based [22,23]. Some of the existing works are using these technologies to achieve and improve the data provenance as different research has been carried out in this direction [24,25,26,27]. We considered different healthcare applications of data provenance proposed by authors such as electronic health record sharing, personal health data, electronic mobile health applications, COVID vaccination data, and electronic patient data.

After 2018, GDPR compliance is an important aspect. There is a need to explore GDPR compliance in the existing research for data provenance in the healthcare sector. Because data provenance consists of sensitive information, it belongs to patients in the healthcare system. For EU citizens, there was a data protection act in 1995 which was replaced by the General Data Protection Regulation (GDPR) in 2018 [28]. The purpose was to protect EU citizens’ rights and to make companies comply with regulations according to GDPR rules [29]. This opens a new direction for researchers, and very little effort is made in this respect.

The purpose of the SLR is to focus on data provenance in healthcare data and perform an extensive literature review on existing techniques for achieving provenance and existing related work in the healthcare domain to find open research issues for future research directions. The aim and objective of this SLR is:

- To study the state-of-the-art technologies used for data provenance in healthcare.
- To provide insight to readers about important attributes of data provenance for healthcare applications.
- To identify the latest trend to achieve data provenance.
- To identify research gaps and provide future research directions.

We used the methodology set by Kitchenham and Charters [30] to conduct this SLR. After careful analysis of research articles, inclusion and exclusion criteria were applied. We considered research articles according to the research questions. We answer the research questions based on an in-depth analysis of these research articles. This helped us to identify potential research gaps, and researchers can provide solutions in their future research.

The rest of the paper is organized as follows: Section 2.1 provides the data provenance overview, Section 2.2 provides the healthcare overview, Section 2.3 describes healthcare and data provenance to understand the requirements of provenance in the healthcare field, and Section 2.4 and Section 3 show the proposed methodology for conducting an SLR related to data provenance in healthcare. The results and discussion are provided in Section 4 and Section 5. The conclusion is provided in Section 7.

## 2. Background

This section introduces data provenance and healthcare data. Then, the importance of data provenance in healthcare is discussed. 

### 2.1. Data Provenance Overview

In this section, we introduce the basic concepts of data provenance, its main usages, implementation technologies, and the basic architecture of data provenance systems.

#### 2.1.1. Data Provenance

Different authors have defined data provenance in different ways [31]. According to some researchers, it is defined as the origin or source of data [1], while others define [32] data provenance as tracing the source of data and how and from where data came. According to authors in [33,34,35], data provenance is defined as collecting the whole process of information from the generation and evolution of data over time. The authors in [36] defined data provenance in terms of healthcare data as follows: “Provenance is defined as attributes about the origin of health information at the time it is first created and tracks the uses and permutations of the health information over its lifecycle”.

#### 2.1.2. Usage of Data Provenance

With the help of provenance information, we can find the origin of data and the operations performed on data at different stages and times. Data provenance has been applied in different domains [34]. There are different usages of data provenance in different domains. A list of data provenance usage is provided in Table 1.

#### 2.1.3. Short Description of Technologies Used for Data Provenance

**Figure 1:**
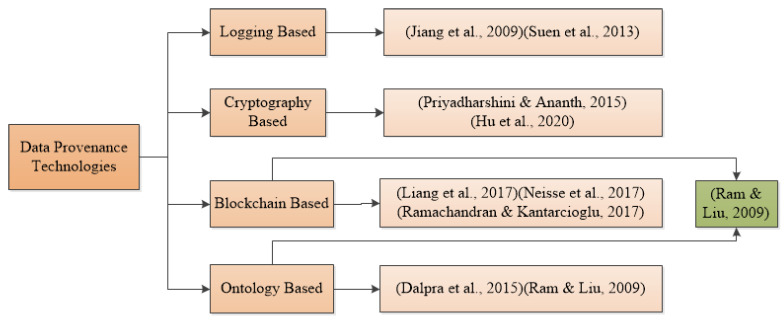
Data provenance technologies [14,15,16,20,21,23,31,34,40].

Currently, there are four types of technologies [34,37,38] available for data provenance. The names for these technologies and short descriptions are provided in Figure 1 and in the sub-section. These technologies can achieve data provenance with the help of each other. The authors in [39] achieved provenance in the supply chain using ontology-based blockchain systems. More details are available in the upcoming section.

(a) Logging-based technology

Logs have pivotal significance in data provenance as they furnish an intricate account of events and activities transpiring within a system or application. Logs play a crucial role in capturing the history and lineage of data within a system or application. They provide a detailed record of events, actions, and interactions related to the data, including their creation, modification, movement, and access. The problems associated with available logs can be resolved much faster compared to those lacking attached logs [14]. Traditional log management systems store the logs in just one node. This limitation can be overcome using an end-to-end provenance mechanism as proposed by [15]. Logs contribute to data provenance through providing a comprehensive record of data-related events, enabling traceability, accountability, auditing, incident investigation, problem diagnosis, and performance optimization.

(b) Cryptography-based technology

Digital signature and Message authentication code are the cryptographic mechanisms [16]. With the help of these techniques, one can find the origin of data. The problem with cryptographic technology is that it did not provide data processing history [34]. It is important to note that while cryptographic techniques contribute to data provenance through addressing security and integrity aspects, achieving comprehensive data provenance may require additional measures such as metadata capture, audit trails, and logging mechanisms.

(c) Blockchain-based technology

Data provenance in the blockchain can be achieved through blockchain transactions that record data operations [19]. Blockchain-based provenance examples include provchain [20]. Some authors achieved provenance using smart contracts [21,40]. These approaches help in achieving data provenance using blockchain.

(d) Ontology-based technology

In the computer science community, ontology-based research has achieved tremendous attention in the field of databases, artificial intelligence, and computational linguistics [22]. For ontology-based provenance, there exist some ontologies [23,31].

#### 2.1.4. General Architecture for Data Provenance

**Figure 2:**
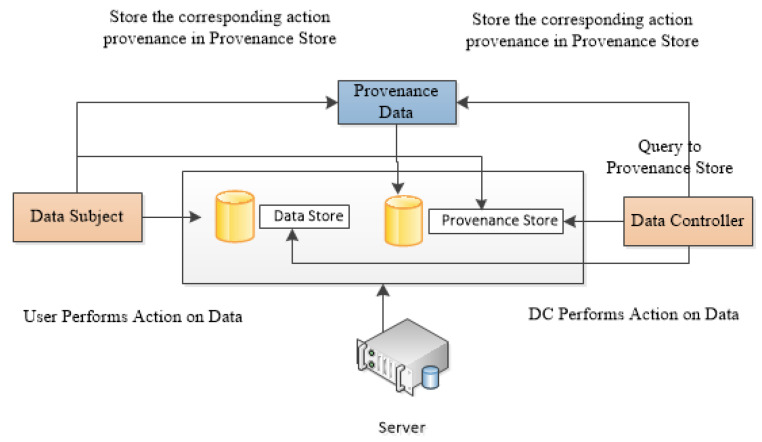
General data provenance architecture.

The general architecture in Figure 2 explains that users (data subjects) can perform actions on data stored on the server. Whenever the user takes an action on the data, their corresponding provenance data will be stored in the provenance store. In the same fashion, whenever the data controller takes any action on the data, their corresponding provenance data will also be stored in the provenance store. Similarly, if the data controller wants to share the data with any third party, we can store the provenance data for user trust and satisfaction. The data controller can query the provenance store and may obtain the corresponding results.

### 2.2. Healthcare Overview

In this section, we have provided an overview of healthcare data, the role of healthcare systems, healthcare in terms of the World Health Organization (WHO), and the typical architecture of the healthcare system.

#### 2.2.1. Healthcare Data

Electronic health systems or digital healthcare systems consist of healthcare data and are implemented all over the world. According to the European Health Digital Service Infrastructure, patients are treated positively using healthcare data [36]. Patients have electronic health records [41] in developed and developing countries. Researchers of healthcare big data in the world are struggling to deal with the multidimensional nature of healthcare data. Also, the sharing of healthcare-sensitive data is a challenge, and data providers hesitate while doing this [42]. Researchers, analysts, patients, and doctors want to access healthcare data instantly and data provenance in this situation becomes more important [1].

#### 2.2.2. Healthcare Stakeholders

**Figure 3:**
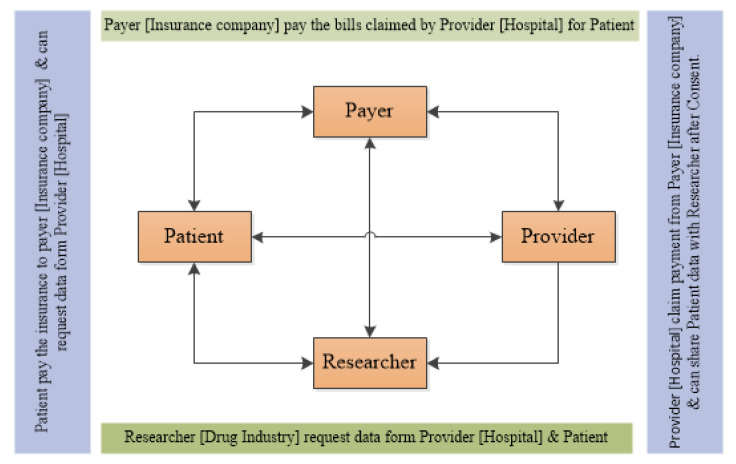
Healthcare data stakeholders.

Currently, there are four stakeholders in healthcare data. These are patients, providers, payers, and providers [42]. Communication and collaboration among these stakeholders are of much importance to making a detailed analysis of patient medical records. These entities are linked with security and privacy threats. Patients produce data and these data are from clinical records or sensors as wearable devices [43]. Payers pay the healthcare cost, i.e., private companies or insurance companies. Medical records are collected and stored by providers. An analysis is performed by researchers and analysts on the data provided by providers to improve the performance of the healthcare industry. Figure 3 shows the relationship between the stakeholders of healthcare data.

There is a need for strong collaboration and communication between the stakeholders of healthcare records. Privacy and security issues are interlinked between these entities because patients are a source of data in healthcare systems. The data are produced using patient medical records and from wearable devices [43]. Payers such as insurance companies, bank lenders, etc. paid the healthcare cost directly or indirectly. Providers collect and store the patient’s health records. A researcher is an entity that collects the record of a patient from a provider with patient consent or directly from the provider.

#### 2.2.3. The WHO and Healthcare

To strengthen the healthcare system, the WHO has devised a health model in terms of six building blocks [44]. The six building blocks are service delivery, health workforce, information, medical products, vaccines and technologies, financing, and leadership/governance [45]. The six building block goals or outcomes are improved health, responsiveness, financial risk protection, and improved efficiency. The WHO’s health system framework with these six building blocks helps in identifying the strengths and weaknesses of any health system in the world [44].

### 2.3. Healthcare and Data Provenance

In this section, we have explained the importance of data provenance in the healthcare system. Though healthcare systems can work alone with data provenance services, they can provide better health services to patients [46]. Also, an example structure of data provenance is provided at the end of this section.

Healthcare data can be used by doctors, researchers, and analysts to achieve their goals. For research purposes, electronic health data offer huge potential for researchers, and computational power is growing day by day with methodological developments that can deal with big data easily [47,48]. The data belong to the patient, and they become risky when shared with doctors, researchers, or analysts. In healthcare, adversaries are always ready to attack patient data. It is always important to record who is accessing the patient’s health data, when, and why. So, provenance becomes critical for patient data safety [36]. Although electronic health record systems define how they work and how they share/exchange patient data [46], they do not discuss, capture, or store the process of information sharing. Here, the provenance system helps in storing or recording the way the data were created or shared with other entities [49]. Data provenance helps in providing the answers to questions about who collected the data, why they collected the data, when they collected the data, and what type or part of the data was collected [37]. 

Table 2 represents an example of a data provenance record consisting of several fields which show how data provenance may look [50]. Each action of the data subject on the data, as depicted in Figure 2, will be stored in the data provenance store with the corresponding action.

### 2.4. GDPR, Healthcare, and Data Provenance

For EU citizens, there was a data protection act in 1995 which was replaced by the General Data Protection Regulation (GDPR) in 2018 [28]. The purpose was to protect EU citizens’ rights and to make companies comply with regulations according to GDPR rules [29]. Citizens are known as data subjects in GDPR terms. The GDPR guides companies on how to use and process data subject data and guides the data subject about how to use and control the data. Because any sort of data like phone number, email address, location, etc. [51] can reveal the identity of a data subject, citizens are very worried about privacy issues due to rapid changes in technology and digitalization in all sectors like healthcare. Healthcare data consist of the medical history of a patient [52]. According to GDPR Article 9, health data are a special category of data. 

There is a need to develop GDPR-compliant healthcare systems with data provenance. There exists prior literature about the GDPR articles. The authors in [53,54] used a logical deletion method to solve the issue of Article 17 of the GDPR. Article 7 of the GDPR emphasizes consent management. Data subject data cannot be stored without prior consent [55] and the authors in [56] proposed a solution according to GDPR compliance. Finding healthcare systems that are complying with GDPR in terms of data provenance is also the focus of this SLR.

## 3. Research Methodology

**Figure 4:**
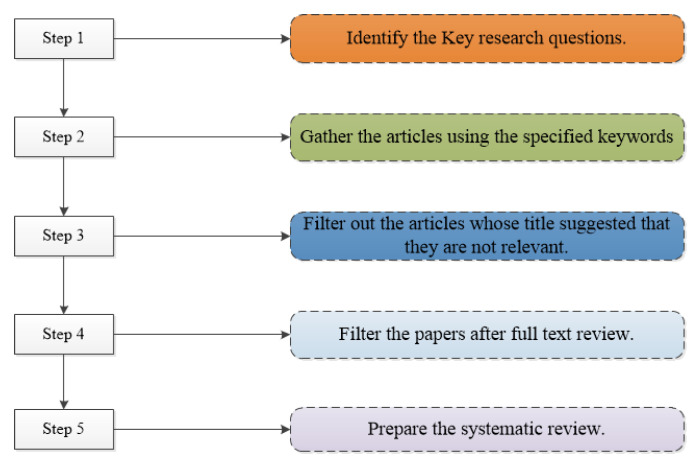
Basic steps in conducting the review.

First, in a systematic literature review (SLR), we have defined specific research questions. It uses a well-defined methodology to answer those questions through collecting, classifying, and extracting all existing research [57,58,59]. The basic steps in conducting any SLR are shown in Figure 4.

Different authors have proposed guidelines for writing a systematic literature review. However, in this SLR, we followed the steps recommended by Barbara Kitchenham [57,58,59]. Many high-impact-factor peer-reviewed journals [60,61] followed this methodology. This process is very famous and specifically designed for conducting systematic reviews in the field of computing research. 

### 3.1. Need of Conducting SLR

According to our knowledge, there is no survey on data provenance in the healthcare domain that describes how existing technologies are used to achieve data provenance. In Section 4, we briefly described the need of conducting SLR because there is always a need to store the actions of the data subject or data controller, as depicted in Figure 2, and provenance can help in various ways, as described in Table 1. Therefore, the applications of healthcare with data provenance features need to be discussed in detail.

### 3.2. Research Question (RQ) and Motivation

**RQ-1**  Which technologies were used for achieving data provenance?
**RQ-2**  Which combinations of technologies were used to achieve data provenance?
**RQ-3**  Which application in healthcare achieves privacy, security, integrity, traceability, unforgeability, and compliance using the technologies discussed in RQ-1 and RQ-2?
**RQ-4**  What are the major challenges and issues for future research?

Motivation for each research question is mentioned in Table 3.

### 3.3. Search Strategy

Pursuing research questions, the following search queries were executed to collect maximum literature for review: “Data Provenance and Healthcare data”, “Data Provenance and Electronic Health Records”, “Blockchain-based Data Provenance”, and “Data Provenance Technologies”. After careful analysis of databases (Google Scholar, ACM, and IEEE Explore), we collected 785 studies.

### 3.4. Inclusion and Exclusion Criteria

**Figure 5:**
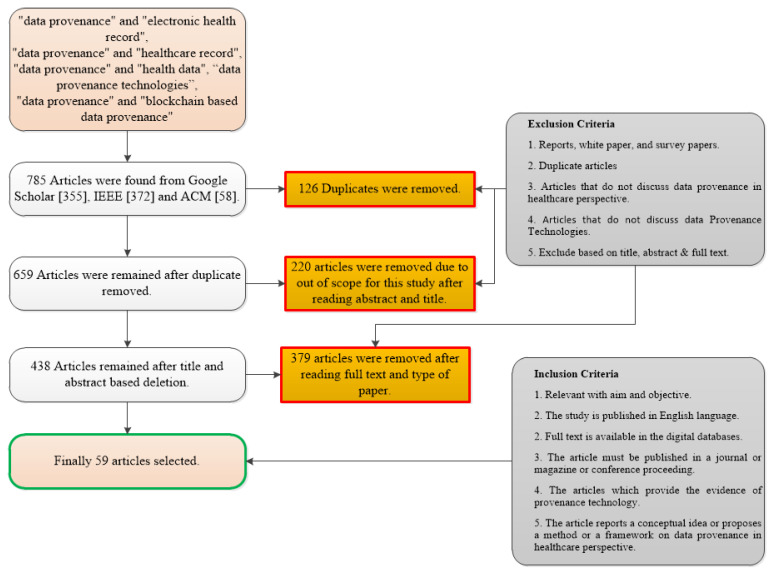
PRISMA chart for SLR.

In this phase, the collected studies were analyzed within the research scope, i.e., data provenance and healthcare. Some studies were found exactly aligned with the research area and some were found partially or completely out of the research scope. The articles were selected based on an outline set by [57]. The following exclusion and inclusion criteria were applied to extract the final list of articles. Figure 5 shows the steps that how inclusion and exclusion criteria are applied in the form of a Preferred Reporting Items for Systematic Reviews and Meta-Analyses (PRISMA) chart. We followed the steps for article selection according to the methodology set by Kitchenham in [57] and used the PRISMA chart for only this particular step of inclusion and exclusion. 

Exclusion criteria:

(a) Reports, white papers, and survey papers;
(b) Duplicate articles;
(c) Articles that do not discuss data provenance from a healthcare perspective;
(d) Exclude based on title, abstract, and full text.

Inclusion criteria:

(a) Relevant with aim and objective;
(b) The study is published in the English language;
(c) Full text is available in a digital database;
(d) The article must be published in a journal or magazine or conference proceeding;
(e) The article reports a conceptual idea or proposes a method or a framework for data provenance from a healthcare perspective.

### 3.5. Classification Criteria

The research questions in this SLR consist of two parts to analyze the existing research for data provenance in the healthcare domain. We classified the shortlisted studies according to the research questions. 

### 3.6. Data Extraction

In the next step, we extracted and analyzed the shortlisted studies to find out the information according to the research questions. For each included study, the data extraction tables were set and filled. Table 4 compares papers for further analysis based on the specific characteristics which are crucial for data provenance. These are discussed below.

#### 3.6.1. Technologies

For data provenance, we found four types of technologies from [34,37,38]. These can also work in combination to achieve data provenance, like in [36,39,62]. We tried to find out the trend in using these technologies for data provenance in the healthcare domain in recent times.

#### 3.6.2. Security

A data provenance system needs to satisfy fundamental security requirements such as confidentiality, integrity [34,50], and availability [34,50,63]. It is crucial to consider the privacy implications as data provenance can potentially expose sensitive information. Therefore, encrypting both the data provenance and the source data becomes essential in ensuring the protection and privacy of the information. Data inside the provenance store must be immutable. There is a need to secure data provenance, which helps in maintaining the confidentiality and integrity of source data, and the security of the provenance is also important. The provenance of data must be available for users at any time from anywhere. In the case of patient data in an emergency case, it is subject to high availability.

#### 3.6.3. Storage

According to the authors in [64], provenance data can be stored both ways, e.g., in centralized and distributed locations. Using a centralized approach, the maintenance is very difficult to manage, whereas in a distributed approach, the cost matters. We have explained this in the Discussion section. 

#### 3.6.4. Metadata

Provenance data are a type of metadata that belongs to an entity and records the creation and usage of data sources, also called lineage or pedigree [33,34]. Provenance metadata are of utmost importance, just like the data itself, because an adversary may use these metadata to perform a privacy attack. Metadata, which describe the data source, demand privacy protection, especially in smart health applications. For example, a patient’s disease history consists of highly sensitive information [65]. There is a need to focus on this aspect of data provenance. 

#### 3.6.5. Overhead

In any application, minimizing overhead is crucial for achieving high performance [66]. It is imperative to ensure that the impact of provenance collection remains minimal [67]. The authors in [66] considered less overhead as a design goal to increase the performance of the proposed system. Through reducing unnecessary overhead, such as computational or resource-intensive processes, the system can operate more efficiently and effectively. There must be low overhead for accessing and storing provenance data. This directly affects the scalability issue because data provenance transactions put an extra burden on the application with source data transactions. There is a need to control this cost. 

#### 3.6.6. Unforgeability

It pertains to the act of falsifying data provenance through manipulating both the source data and introducing counterfeit data. Provenance must be tightly coupled with its source data and the provenance system must detect if an adversary tries to forge any fake data. Someone can forge a medical report to avoid an investigation of misdiagnosis [50]. There is a need for such a system which has the feature of unforgeability. 

#### 3.6.7. Compliance

Various types of data, including phone numbers, email addresses, and location information, have the potential to expose the identity of individuals [51]. With rapid advancements in technology and widespread digitization across sectors like healthcare, citizens have become increasingly concerned about privacy issues. The growing integration of technology and digitalization has heightened these worries, particularly regarding the safeguarding of personal information. Healthcare data consist of the medical history of a patient [52]. There is a need to develop GDPR-compliant healthcare systems with data provenance. In this SLR, we tried to find healthcare systems that are complying with the GDPR in terms of data provenance. Article 30 of the GDPR refers to recording processing activities and Article 32 refers to the storage of processing.

## 4. Results

### 4.1. Research Trend

The findings of the systematic review are summarized in this section. Table 4 shows the number of publications per year from 2010 to 2022 considered for this study. From the table, we observed that there is 1 thesis, 1 book chapter, 24 journals, and 33 conference papers.

**Figure 6:**
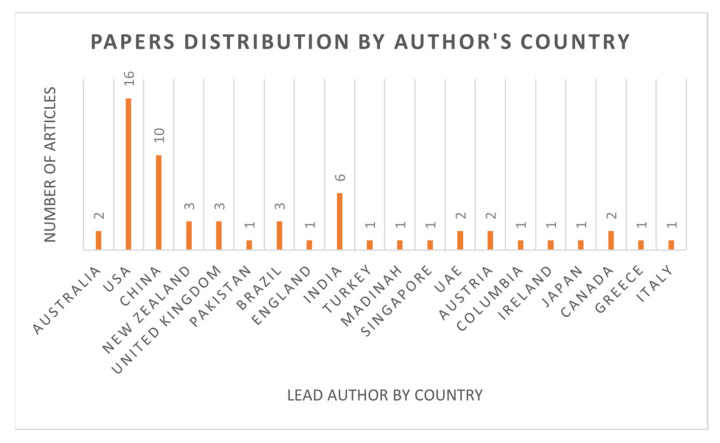
Authors by country.

Figure 6 shows the distribution of papers by author country-wise. Researchers from different countries are working on the problem for which this SLR was conducted. From this figure, we can figure out that most of the affiliations of authors belong to the USA and China.

**Figure 7:**
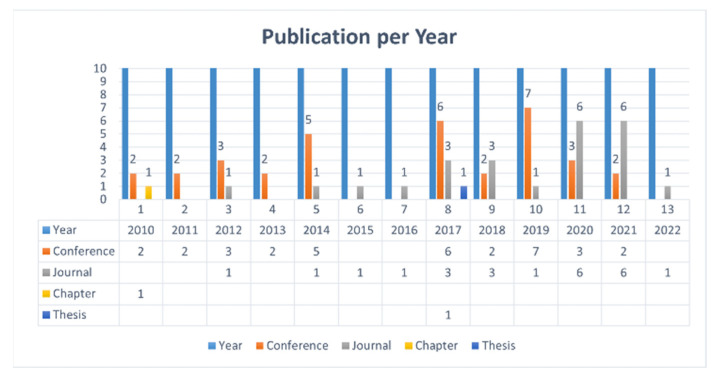
Publication trend over the years.

For observing research trends and impact, the best way is to find the number of publications in a year. There are many research articles published in a year by various publishers. We have identified relevant articles from these publishers according to the research aims and objectives. In Figure 7, we found the publication trend over the years using Table 4. From 2017 to 2021, the research in this direction makes progress. We have shown the importance of the topic in a timeline diagram in Section 5. 

**Figure 8:**
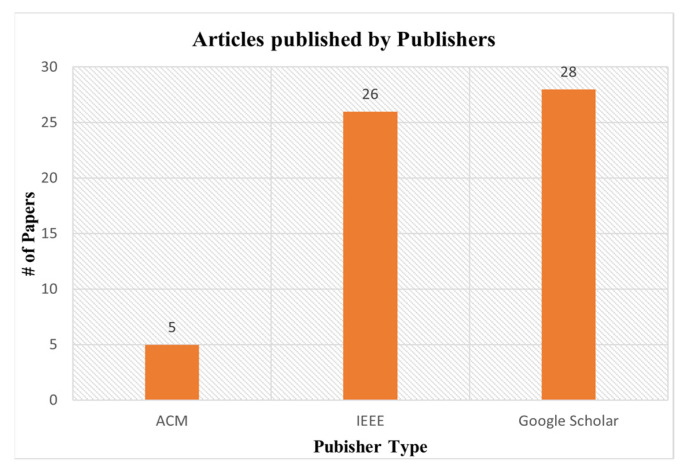
Articles published by publisher type.

**Figure 9:**
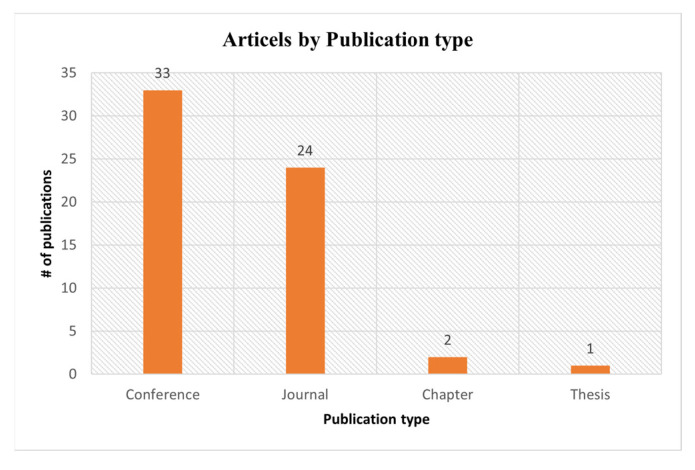
Articles published by various publication types.

The number of articles published by various publishers and publication types are shown in Figure 8 and Figure 9, respectively. We explored three digital libraries (Google Scholar, IEE, and ACM). From the results (as shown in Figure 9), we found that most articles are published in conferences. Most of the papers we selected for this SLR are from Google Scholar. The Google Scholar database shows the research articles published by various publishers. But we also searched two other reliable repositories (ACM and IEEE Explore) for this SLR. 

### 4.2. Research Questions

In this section, we utilize our findings to cross-examine the research questions we have identified in Section 4. We provide a discussion of our analysis and address the limitations of the review.

#### 4.2.1. Addressing RQ-1

The first research question considered for this study was “Which technologies were used for achieving data provenance?” In Section 2, Figure 1, the names of the technologies are provided with a brief description. Now, we briefly present these technologies and their purpose in Table 5. That is how different research works used these technologies.

#### 4.2.2. Addressing RQ-2

The second research question considered for this study concerned “the combination of technologies that were used for achieving data provenance”. In Section 2, Figure 1, an example is given in the figure which shows the combination of technologies, e.g., blockchain and ontology-based combination, for achieving the data provenance. Here, we have presented a combination of technologies and their purpose in Table 6 that explains how different research works use these technologies and achieve their goal. The purpose of this table is to bring the attention of researchers toward achieving data provenance using different combinations of technologies.

#### 4.2.3. Addressing RQ-3

The third RQ for this study is “Which healthcare application achieves provenance security, confidentiality, integrity, availability, metadata protection, scalability, provenance overhead, unforgeability, and GDPR compliance?” We answer these attributes using Yes ‘Y’/No ‘N’ and * which means partially satisfied. Also, maturity level ‘ML’ is considered in this question as an additional attribute. The maturity level can be architecture ‘A’, proposed ‘P’, implemented ‘I’, or evaluated ‘E’. We summarize the results in Table 7 based on these data-provenance-related attributes. After creating Table 7 for a comparison for existing research, we discuss the attributes in the next discussion sections.

#### 4.2.4. Addressing RQ-4

RQ-4: What are the major research gaps in the domain of data provenance in healthcare systems?

In previous questions, we discussed the technologies used for data provenance and healthcare systems/applications which achieved the data provenance. In this section, the most important question “Finding the Research gap” is addressed. Although a lot of work is done in this field, there are still research gaps that need to be filled. The most important research gaps are as follows:

(a) When the provenance is stored, the occupation of the data subject is not considered. Some jobs are highly sensitive, e.g., civil servants, government employees, armed forces, researchers who work in atomic plants, etc., and others have less sensitive information. We have found in our SLR that provenance is not classified and prioritized according to its sensitivity.
(b) Provenance metadata protection is not discussed in detail in the literature. Metadata consist of sensitive and non-sensitive attributes, and adversaries can attack using non-sensitive metadata. The classification between sensitive and non-sensitive metadata stored in provenance is very important for user privacy protection. This can be addressed by researchers in the future.
(c) The provenance storage of data subjects is directly related to privacy. There is a need to perform plenty of work in this regard. If the GDPR rules are wrapped into provenance data, privacy may be preserved. In our SLR, we have found a few research articles that emphasize the importance of the GDPR as it relates to healthcare data provenance. There is a need to address this issue not only on a conceptual level but also in an implementation context.
(d) The GDPR highlights the importance of the data subject’s consent for data handling. Unfortunately, considerable work is not carried out for healthcare data provenance.
(e) Current research is achieving data provenance using blockchain technology. A public blockchain has issues with GDPR compliance and scalability. The existing web-based or cloud services-based healthcare systems deal with GDPR and scalability issues efficiently but are susceptible to a singular point of failure. The combination of web, cloud, and blockchain, either public or private-based approaches, may solve the issues of GDPR compliance and scalability.

## 5. Discussion

In this section, we have presented our timeline and taxonomy of data provenance in the healthcare domain to uncover and analyze them easily. We considered some important features and technologies which are important to achieve provenance and provide a comparison in Table 7. The techniques are log-based, cryptography-based, ontology-based, and blockchain-based. The essential requirements of data provenance are provenance security, compliance, storage, unforgeability, overhead, revocability, and metadata protection. 

### 5.1. Taxonomy

**Figure 10:**
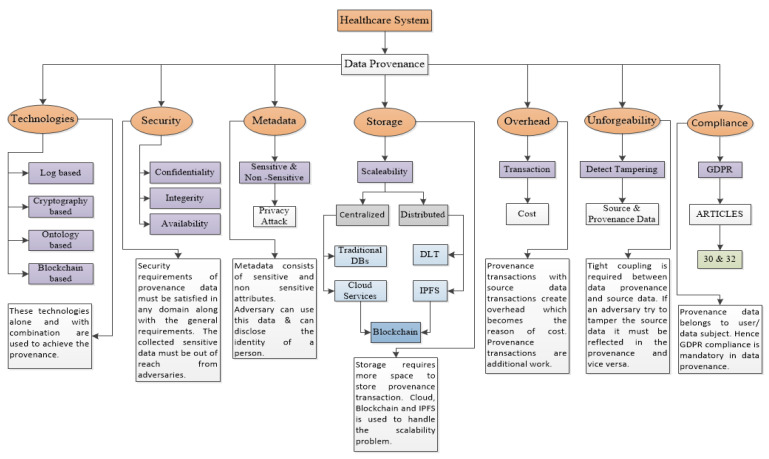
Taxonomy of data provenance in healthcare.

Figure 10 presents the taxonomy of data provenance in healthcare. Requirements for data provenance and research work carried out are explained in the following sub-sections. 

#### 5.1.1. Technologies

During our investigation of data provenance, we identified four distinct types of technologies [34,37]. Figure 1 depicts some of the author’s work using these technologies to achieve provenance. These technologies can also be used in combination to achieve data provenance, like in [36,39,62]. The authors in [14,15] proposed log-based provenance. The authors in [16,18,34] used cryptography-based solutions for achieving provenance. For ontology-based provenance, there exist some ontologies [23,31]. Data provenance in the blockchain can be achieved through blockchain transactions that record data operations [19]. One blockchain-based provenance example is provchain [20]. Some authors achieved provenance using smart contracts [21,40]. These approaches help in achieving data provenance using blockchain. We found that, in recent years, the trend is toward using blockchain technology to achieve data provenance. It provides many built-in features required for provenance and it can work well with many existing technologies.

#### 5.1.2. Security

A data provenance system must fulfill general security requirements, e.g., confidentiality, integrity [34,50], and availability [15,34,63]. Data provenance may reveal private information. So, encryption of data provenance is very important with source data encryption. Queries of data provenance and the results of queries in response in any data provenance system must be secure and must not reveal any sensitive information. Data inside the provenance store must be immutable. We found that a blockchain-based solution for data provenance helps in maintaining the integrity of not only source data but provenance, too. In the end, the provenance of data must be available for users at any time from anywhere. In the case of patient data in an emergency case, they are subject to high availability. We found some existing research works [50,94,96,98,102] which are meeting these requirements in their work. In [7,66], the authors discussed the security of provenance but did not individually mention these requirements. 

#### 5.1.3. Storage

The authors in [64] state that provenance data can be stored in either a centralized or distributed manner, offering flexibility in the storage approach. Commonly, storage scalability is measured in terms of performance and capacity. Though blockchain technology is effective at meeting data provenance requirements, it is very hard, due to systematic design, to search and query provenance information. But when we use centralized approaches, the maintenance is very difficult to manage. Both approaches have advantages and disadvantages. The authors in [104] said that scalability refers to metadata capturing. It is very important how much metadata are captured in provenance to manage storage scalability. In the taxonomy, we highlighted in the end that mostly the authors [62,96,105] are using blockchain with cloud and IPFS to manage this issue. 

#### 5.1.4. Metadata

Provenance data, also known as lineage or pedigree, refer to a specific kind of metadata that pertain to an entity. They document the origin and utilization of a data source [33,34]. The significance of provenance metadata surpasses that of the actual data, as it can potentially be exploited by malicious actors to carry out privacy breaches. In particular, metadata that characterizes the patient as the data source requires enhanced privacy protection, especially in the context of smart health applications. The authors in [96] proposed a solution based on blockchain technology that captures and stores provenance metadata on the blockchain using smart contracts. They store the data off-chain and store the corresponding provenance on the chain. Metadata consist of sensitive and non-sensitive attributes, and the GDPR has a clear distinction between sensitive and non-sensitive personal data. Gender, date of birth, place of birth, postcode, etc. can disclose the privacy of the data subject. In the existing research trending in healthcare systems related to data provenance, we did not find special attention to the issue of sensitive and non-sensitive data in metadata. There is a need to create such information systems in which data provenance must be stored and classified according to the sensitivity of the metadata. For example, a patient’s disease history consists of highly sensitive information [65]. It is also important for the recent trend of storing provenance metadata in the blockchain.

#### 5.1.5. Overhead

Less overhead is important for high performance in any application [66]. The impact of provenance collection must be minor [67]. In their study, the authors prioritized minimizing overhead as a design objective to enhance the performance of the proposed system [66]. The authors in [50] proposed a cloud-based data provenance system where they discussed the storage and performance overhead and how to optimize them. In a blockchain-based system, there must be low overhead for accessing and storing provenance data. This directly affects the scalability issue because data provenance transactions put an extra burden on the application with source data transactions. Blockchain-based approaches used for data provenance pay the cost of overhead due to provenance transactions. There is a need to control this cost. 

#### 5.1.6. Unforgeability

It refers to the forging of data provenance with fake data as well as with source data. Provenance must be tightly coupled with its source data, and the provenance system must detect if an adversary tries to forge fake data. Someone can forge a medical report to avoid an investigation of misdiagnosis [50]. So, the proposed system must have this feature of unforgeability. The authors in [106,107] presented protocols for forgeability in cloud-based systems. In the healthcare domain, very few authors [36,94,98] have discussed and fulfilled this requirement. 

#### 5.1.7. Compliance

Various types of information such as phone numbers, email addresses, and locations have the potential to unveil the identity of an individual. Swift advancements in technology and the widespread digitization across various sectors, including healthcare, have caused significant concerns among citizens regarding their privacy. The medical records of patients make up healthcare data [52], which is a crucial component in this context. According to GDPR Article 9, health data are a special category of data. There is a need to develop GDPR-compliant healthcare systems which also capture provenance. There is ongoing work to develop healthcare systems that are GDPR-compliant. The authors in [53,54] used a logical deletion method to solve the issue of Article 17 of the GDPR. Article 7 of the GDPR emphasizes consent management. A data subject’s data cannot be stored without prior consent [55], and the authors in [56] proposed a solution according to GDPR compliance. In this SLR, one of our focuses is to find healthcare systems that are complying with GDPR in terms of data provenance. Article 30 of GDPR belongs to recording processing activities and Article 32 belongs to the storage of processing. 

We found no work in which the authors discussed these articles in existing research on achieving provenance in healthcare systems. Keeping compliance in mind while developing provenance systems may open new directions.

### 5.2. Timeline

**Figure 11:**
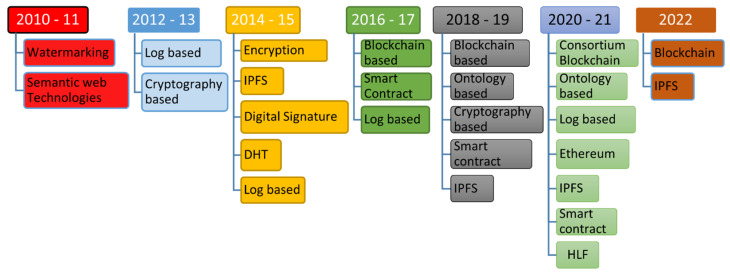
Timeline of technologies used for data provenance in healthcare.

We have analyzed four basic technologies to achieve data provenance and listed them in the taxonomy. We also figured out the ongoing trend of the research community through observing their interests in achieving data provenance using these technologies. The timeline depicts the interest of the research community in a graphical view to more easily understand other researchers. In Figure 11, the timeline is presented from 2010 to 2022, where the technologies are mapped alongside the years. On the *X*-axis, the years are plotted, and technologies are plotted on the *Y*-axis. We created the timeline through keeping our focus on the healthcare domain. In 2006, authors argued that traditional log recording is suited for a single-node system [30]. Though the limitations were overcome by authors in [15], the report of authors in a survey-based study presented that log-file-based provenance is not suited to the distributed nature of current systems [34]. We found a log-based approach from 2012 to 2017 for healthcare domain data provenance. Then, the trend shifts toward other techniques. In 2020, the authors [73] proposed an approach with log files with a distributed ledger to achieve data provenance. Their effort demonstrated that log-based techniques may be helpful in the future to work in combination with other technologies. Cryptography-based technologies are always in use for data provenance and work with other technologies for data provenance. This technology helps in identifying the origin of data efficiently [108]. Watermarking [18], encryption [66], IPFS [62,102], digital signatures [16,66], DHT [105], etc. are all based on cryptography. But, in the case of data processing history, this technology cannot store records [1]. From 2010 to 2022, we have found this technology helpful in data provenance. From 2018 to 2021, ontology-based provenance was used in the healthcare domain. Capturing provenance using ontology is very flexible and can cover as many domains as possible [31,109]. It is also very helpful in provenance analysis [110]. Several ontologies exist in the literature for data provenance. Ontologies store the provenance record [111] and there is always a need to apply a technique on ontology to protect it from malicious entities. From all these technologies we found that the adoption of blockchain technology is growing day by day due to its features for achieving data provenance. From 2016 to 2022, the authors adopted blockchain technology. In research question 3, we listed several papers in which authors proposed and implemented blockchain-based solutions in combination with IPFS [62,102], smart contracts [83,101], and the PROV model [36]. This technology is very useful in distributed-nature application scenarios [32,33]. It is very complex in terms of implementation context [1]. Also, scalability is a challenge for this technology. In the taxonomy, we have highlighted the authors [62,96,105] who are using blockchain with cloud and IPFS to manage this issue. 

## 6. Contributions

**Figure 12:**
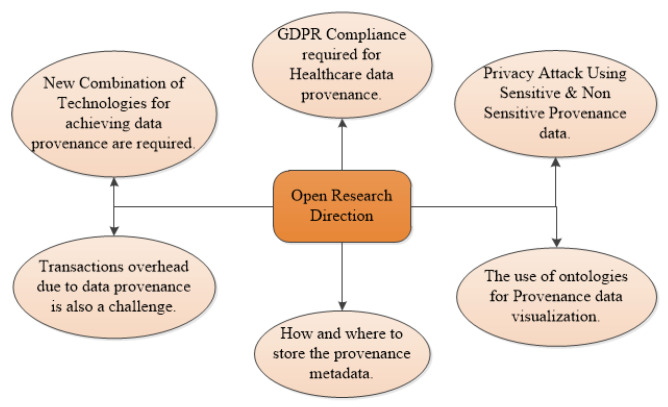
Open research directions.

In this SLR, we have explored the research work performed for achieving provenance in the healthcare domain. We have discussed the technologies used to achieve provenance in prior literature. We have also identified the research gaps based on the SLR, and based on our findings we have also made several suggestions for future research directions. Figure 12 presents the open research directions. The implications are as follows.

1. This paper presents the techniques used to achieve data provenance in healthcare. We found four techniques, i.e., log-based, cryptography-based, blockchain-based, and ontology-based techniques for achieving data provenance, as discussed in Table 5. Further, we explored the work carried out to achieve the data provenance with the combination of these techniques, as discussed in Table 6. We found that limited work was performed to achieve provenance using the combination of these technologies. Researchers can find possible solutions using these techniques, as discussed in Table 6 or can introduce new techniques to achieve data provenance.
2. This SLR discussed the GDPR compliance issue in the field of healthcare data provenance, which was not discussed in any other SLR. In Table 7, we have explored different attributes. GDPR compliance is one of them, and the purpose was to explore compliance issues because provenance data belongs to entities, i.e., the data subject who is a patient in case of healthcare data, or the data controller, etc. GDPR Article 30 is related to the record of processing activities. In data provenance, we store actions performed by the data subject or data controller to achieve trustworthiness. Though the authors, in their research work, achieved data provenance, they did not discuss the GDPR compliance issues. Very rare work is done in this regard. Dealing with the GDPR compliance issues when achieving data provenance in healthcare will help and set a new direction for research in the future.
3. In existing healthcare systems, authors have achieved data provenance. But they did not explain which types of data they are storing during the operations or actions performed by the user or administrator. Because provenance consists of sensitive and non-sensitive information, an adversary can attack and may reveal the identity of the user. So, when you are collecting provenance, it is important to classify the metadata into different categories and protect them from adversaries using encryption techniques. Provenance security and provenance metadata are very important attributes from the research point of view in the future.
4. Blockchain technology ensures the immutability of data. But when dealing with big data, especially in the medical field, it becomes challenging. As the data grow in the blockchain, the performance of the application degrades. Every new miner will download a complete copy of the data. Also, the GDPR conflicts with blockchain for storing data. The data stored on a blockchain cannot be easily modified or deleted; it can create compliance issues under the GDPR. For big data applications in the future, we recommend using cloud services with blockchain technology. Through integrating blockchain and cloud services, organizations can leverage the strengths of both technologies to achieve a more comprehensive solution. Blockchain will deal with security and the cost of maintenance, and the cloud environment will deal with storage issues, performance, and compliance. Its decentralized and tamper-resistant nature enhances data security, making it difficult for unauthorized individuals to manipulate or tamper with the stored information.
5. The timeline in Figure 11 shows that the trend in achieving data provenance after 2015 is by means of blockchain technology. Blockchain works using transactions. The transactions become the reason for communication overhead in the blockchain as the network grows. The provenance may put an additional burden on the blockchain due to additional transactions of data provenance in terms of cost. The evaluation of data provenance transactions with data storage is also required.
6. Another important direction that is already in use for capturing provenance is the use of ontologies. Capturing provenance using ontology is very flexible and can cover as many domains as possible. Several ontologies exist in the literature for data provenance. It is also very helpful in provenance visualization and analysis. Ontologies store the provenance record, and researchers can apply encryption techniques to provenance data in an ontology to protect it from malicious entities. In ontologies, one can classify the data in different classes according to the importance of data.

### Limitations

Despite the various benefits of conducting a systematic analysis, certain drawbacks need to be considered, e.g., bias in the collection, publication, the extraction of data, and misclassification [30]. During the SLR, we found many domains in which data provenance exists. The focus of this SLR was purely limited to research articles that only addressed data provenance for healthcare data and GDPR compliance. We have developed an efficient research protocol to achieve the maximum possible number of papers. Inclusion and exclusion criteria were set to ensure that the focus was on the research topic.

## 7. Conclusions and Future Work

The SLR answers the questions set in the research methodology of the current state of the art in data provenance in the healthcare domain. We have discussed the types of data provenance and the technologies used to achieve it in the healthcare domain. We have identified blockchain technology as the latest technology in healthcare systems to achieve immutable data provenance. Our findings show that several challenges still require more research, i.e., provenance security, provenance communication overhead, scalability, revocation, and GDPR compliance. Very little effort is made on GDPR compliance data provenance in the literature. 

In the future, this SLR may assist as a reference in this field. Potential researchers, with the aid of our contributions and identification of research gaps, may design a new model or architecture. The use of blockchain technology with existing data-provenance-based healthcare systems in particular may overcome the existing problems with more exciting solutions.

## Figures and Tables

**Table 1:** Usages of data provenance.

Sr #	Usage	Ref.
1	Ensure data trustworthiness and data quality.	[3]
2	Detect errors in data generation and processing.	[4,5,6]
3	Help in data recovery and trust management between sensors.	[7]
4	Improve data readability and describe data citation.	[8]
5	Useful in forensic investigation in the field of IoT healthcare.	[9]
6	In healthcare, the complete information of healthcare creation, access, and transfer.	[10]
7	Achieving trust using data provenance for cloud provider.	[11]
8	In home care, why and how procedures applied in treatment can be improved.	[12]
9	Trusted document history of artwork.	[13]

**Table 2:** Structure of data provenance entry.

#	ID	Date	Time	Action			Revision	Hash
				Name	Reason	Location		
1	11	25 May 2022	12:40:22	Create	Blood Test	Florida	0	X
2	12	26 May 2022	14:23:01	Append	Medical Report	Texas	1	Y
3	-	-	-	-	-	-	-	-

**Table 3:** Research questions and motivation.

RQ	Motivation
1	The objective of this research question is to highlight the technologies which achieve provenance.
2	The objective of this research question is to find the technologies which achieve data provenance in combination with each other.
3	The objective of this research question is to find healthcare applications that achieve data provenance while maintaining privacy, security, integrity, traceability, unforgeability, and compliance.
4	The objective of this question is to find out those data provenance challenges and issues which need to be addressed in the future.

**Table 4:** Articles published by various publishers.

Article Type	2010	2011	2012	2013	2014	2015	2016	2017	2018	2019	2020	2021	2022
Conference	2	2	3	2	5			6	2	7	3	2	
Journal			1		1	1	1	3	3	1	6	6	1
Chapter	1												
Thesis								1					

**Table 5:** Data provenance techniques and their purpose in different research works.

Ref.	Provenance Technology	Purpose
[10]	Blockchain-based	The authors proposed a generic framework based on blockchain which is simplified in adoption and provides data provenance.
[15]	Log-based	The authors proposed a data event logging system in the cloud which captures, analyzes, and visualizes data events. It deals with cloud security problems related to critical data.
[16]	Cryptography-based	The authors in this work propose an approach called cryptographic provenance verification consisting of two modules: sign and verify. The purpose was to protect data from fake keystroke injection.
[17]	Cryptography-based	The author in this technique proposed a watermarking-based method for provenance embedding to avoid insider and outsider attacks.
[20]	Blockchain-based	The purpose of the authors was to achieve data provenance through detecting operations on cloud files as objects with the help of blockchain technology. They store the provenance in blockchain transactions.
[23]	Ontology-based	The authors in this work presented a proposal using the PROV process based on PROV-O ontology to capture the process data provenance in software processes to improve product quality.
[31]	Ontology-based	The authors proposed a w7 model which captures the provenance semantics for data in any domain. The purpose was to capture domain-specific provenance.
[68]	Log-based	Using logs, the authors proposed a model for identifying and collecting provenance data from log files and showed that capturing data provenance through logs is very helpful in some circumstances.
[69]	Log-based	According to the authors, this research deals with data security and data activity audit. It was applied to a cloud system.
[70]	Log-based	In this work, the author extracts data provenance through reconstructing log files. He models the information from log files into provenance relations.
[71]	Log-based	In this work, the authors stored provenance data as a separate file and used this provenance to detect changes in the dataset.
[72]	Log-based	The authors proposed a Prov-Trust system that stores the provenance using log files which capture the events of smart contracts or via blockchain transactions, depending on the provenance change event.
[73]	Log-based	The authors proposed a lightweight provenance tracing system based on system event logging and unit-level taint propagation.
[74]	Cryptography-based	Using advanced cryptography algorithms, the authors proposed a data provenance system for secure hosts.
[75]	Cryptography-based	In this paper, the authors proposed a lightweight novel scheme to securely transmit sensor data provenance. This scheme avoids the tampering of data from adversary attacks.
[76]	Cryptography-based	The authors in this research work provided efficient techniques for provenance encoding. This technique is based on a dynamic Bayesian network.
[77]	Cryptography-based	According to the authors, the proposed scheme reduced the storage requirement and computational time for the Internet of Things.
[78]	Cryptography-based	In this paper, the authors proposed a scheme to trace the origin and transformation history of multimedia data sharing and dissemination.
[79]	Blockchain-based	The authors in this research achieve data provenance and data integrity using blockchain with smart contracts.
[80]	Blockchain-based	The authors identified the functional and non-functional requirements for a secure data provenance framework in IoT. They also proposed a solution for scalability and privacy issues in the blockchain.
[81]	Blockchain-based	The author proposed cloud-based data provenance using blockchain. This work traces operations on data and generates provenance. Due to the global blockchain network, tampering with provenance is challenging.
[82]	Blockchain-based	The authors proposed a blockchain-based approach with smart contracts for drug traceability in healthcare.
[83]	Blockchain-based	The author proposed a blockchain-based model for data provenance. Also, their purpose was the security and traceability of personal health data with data provenance.
[84]	Ontology-based	The proposed system detects privacy violation to reduce privacy risks in the healthcare domain. It was implemented using semantic technologies.
[85]	Ontology-based	The purpose was to provide a security model for protecting data provenance using semantic web technologies.
[86]	Ontology-based	This research work deals with the source of data published on the web using provenance vocabulary.
[87]	Ontology-based	In this work, the authors proposed the Provenir ontology. It was designed for the origin of genetic data and has similar features to provenance vocabulary [86].
[88,89]	Blockchain-based	According to the authors, the origins of assets are traceable (like patient records) using blockchain technology and records can be confirmed.

**Table 6:** Combinations of technologies to achieve data provenance.

Ref.	Combination of Technologies	Purpose
[24]	Blockchain- and cryptography-based	The purpose of the author in this research is to store the provenance data efficiently using blockchain and IPFS [25] technology with the secure hash function SHA-256.
[26]	Blockchain- and ontology-based	The author used blockchain and PROV ontology specifications for software provenance.
[27]	Blockchain- and ontology-based	The author of this paper used the ontology and blockchain combination to control the flow of personal data. The use of ontology helps in identifying the entities involved in personal data processing.
[36]	Blockchain and ontology-based	In this research work, the authors solved the problem of data provenance using blockchain and PROV ontology.
[39]	Blockchain- and ontology-based	In this paper, to determine the provenance, the author used the traceability ontology and enforced traceability constraints on platforms based on the Ethereum blockchain.
[90]	Blockchain- and cryptography-based	In this paper, the author stored the cryptographic hash of device metadata in a blockchain and stored the actual data in the cloud for scalability.

**Table 7:** Comparison of research articles.

Ref.	Applied Field	Technologies Used	ML	PS	C	I	A	MP	S	PO	U	GDPR
[91]	Brazilian hemotherapy	Provenance data model	I	N	Y	N	N	N	N	N	N	N
[92]	Healthcare data	HLF, BC, and smart contract	I	Y	Y	Y	Y	N	N	N	N	N
[93]	Medical data sharing	BC and smart contract	I	Y	Y	Y	Y	N	Y	Y	N	N
[94]	Mobile health data	BC and trusted execution environment	E	Y	Y	Y	Y	Y	N	Y	Y	N
[95]	E-health system	BC	P	Y	N	Y	N	N	Y	N	N	N
[18]	Electronic medical record	Digital watermarking	I	Y	N	Y	N	N	N	N	N	N
[36]	Healthcare data	PROV and Blockchain	I	Y	Y	Y	Y	N	Y	N	Y	N
[50]	Healthcare scenario	Not mentioned	P	Y	Y	Y	Y	N	Y	Y	Y	N
[62]	Controlled medication	Ethereum BC, smart contract, and IPFS	I	Y	Y	Y	Y	Y	Y	Y	N	N
[66]	Big data	Digital signature and encryption	P	Y	N	N	N	N	Y	Y	N	N
[96]	Healthcare	Blockchain	P	Y	Y	Y	Y	Y	Y	N	N	N
[7]	IoT healthcare application	-	A	Y	N	N	N	N	N	N	N	N
[80]	Health monitoring system	BC and smart contract	E	Y	Y	Y	Y	N	Y	Y	Y	N
[82]	Health supply chain	Blockchain, Ethereum, smart contract	I	N	N	Y	Y	N	*	N	N	N
[84]	Healthcare data	Semantic web technologies	I	Y	Y	Y	Y	N	N	N	N	N
[85]	Healthcare data	Semantic web technologies	I	Y	Y	Y	N	N	N	N	N	N
[97]	Mobile-based health data	WebView, XCode	E	Y	N	Y	N		N	Y	N	N
[98]	E-health	Blockchain	I	Y	Y	Y	Y	N	N	N	Y	N
[99]	E-health data	Not mentioned	I	N	N	N	N	N	N	N	N	N
[100]	Healthcare	Blockchain, smart contract	I	N	Y	Y	Y	N	*	N	N	N
[101]	Patient data	Consortium BC	I	Y	Y	Y	Y	N	N	N	N	N
[102]	COVID vaccination	Blockchain and smart contract, IPFS	I	Y	Y	Y	Y	N	N	N	N	N
[103]	Medical data	RDBMS	P	Y	N	N	N	N	N	N	N	N
